# Gastrointestinal Stromal Tumor of the Pancreas With Hepatic Metastasis Presenting as Obstructive Jaundice

**DOI:** 10.7759/cureus.90277

**Published:** 2025-08-17

**Authors:** Fahad S Alanazi, Khaled AlQahtani, Abdulkreem Abdulaziz Alnasser, Shakir Bakkari

**Affiliations:** 1 Department of Internal Medicine, King Saud Medical City, Riyadh, SAU; 2 Department of Gastroenterology and Hepatology, King Saud Medical City, Riyadh, SAU

**Keywords:** extraintestinal gist, gastrointestinal stromal tumor (gist), metastatic pancreatic mass, obstructive jaundice, pancreas

## Abstract

Pancreatic gastrointestinal stromal tumors (GISTs) are rare mesenchymal tumors that present significant diagnostic and treatment challenges due to their unusual location and vague symptoms. This report details an unusual case involving a male in the seventh decade of life who experienced obstructive jaundice resulting from a pancreatic GIST situated in the pancreaticoduodenal groove. Diagnosis was confirmed through imaging, histopathological analysis, and immunohistochemical studies. The patient received treatment with imatinib, which led to notable clinical improvement and considerable tumor reduction.

## Introduction

Gastrointestinal stromal tumors (GISTs) are a type of mesenchymal tumor that can develop anywhere along the gastrointestinal tract, accounting for less than 1% of all gastrointestinal tumors. They can arise throughout the gastrointestinal system, from the esophagus to the anus, and predominantly affect older adults. Most GISTs are found in the stomach (60%-70%), followed by the small intestine (20%-25%), colon and rectum (5%), and esophagus (less than 5%) [1,2]. When these tumors develop outside the gastrointestinal tract, they are referred to as extragastrointestinal stromal tumors (EGISTs), which comprise about 5%-10% of all GISTs. Pancreatic EGISTs are particularly rare [3]. Obstructive jaundice is also an uncommon presentation [4].

These tumors can mimic other pancreatic neoplasms, making comprehensive imaging and histopathological evaluation essential for accurate diagnosis. Diagnosis of GIST typically relies on CD117 (KIT) and DOG-1 staining [5]. In cases of metastatic or unresectable disease, imatinib is the treatment of choice [6,7].

## Case presentation

Clinical history

A male in his seventh decade of life, with a history of hypertension and dilated cardiomyopathy, presented with jaundice for four months.

The jaundice was accompanied by dark urine, upper abdominal pain, and unintentional weight loss. He reported no itching, fever, gastrointestinal bleeding, or family history of cancer.

Examination findings

On physical examination, the patient was icteric, and a non-tender mass was palpated in the right upper quadrant of the abdomen without guarding.

Laboratory findings

Laboratory tests showed a cholestatic pattern, with elevated direct bilirubin and alkaline phosphatase levels. The CA 19-9 level was 1000 U/mL. Other investigations, including complete blood count and renal function tests, were within normal limits (Table 1).

**Table 1:** Patient’s laboratory test results with corresponding reference ranges.

Test	Result	Reference range
Hemoglobin	13.2 g/dl	13-15 g/dl
WBC	6 x 10^9/L	4-10
Platelets	400 x 10^9/L	140-400
International normalized ratio	0.92	0.8-1.3
Prothrombin time	10.64 seconds	10-15 seconds
Activated partial thromboplastin time	25.79 seconds	25-40 seconds
Creatinine	32.63 Umol/L	53-97 Umol/L
Blood urea	5.3 Mmol/L	2.1-7.1 Mmol/L
Potassium	4 Mmol/L	3.5-5.5 Mmol/L
Chloride	104 Mmol/L	98-107 Mmol/L
Sodium	137 Mmol/L	135-145 Mmol/L
Aspartate aminotransferase	68 U/L	11-34 U/L
Alanine aminotransferase	72 U/L	0-55 U/L
Alkaline phosphatase	678 U/L	56-167 U/L
Gamma-glutamyl transferase	457 U/L	12-64 U/L
Total bilirubin	518 Umol/L	5.1-20.5 Umol/L
Direct bilirubin	324 Umol/L	0-8.6 Umol/L
CA 19-9	1000 U/mL	0-37 U/mL

Radiological workup

CT of the Abdomen, Pelvis, and Chest

**Figure 1:**
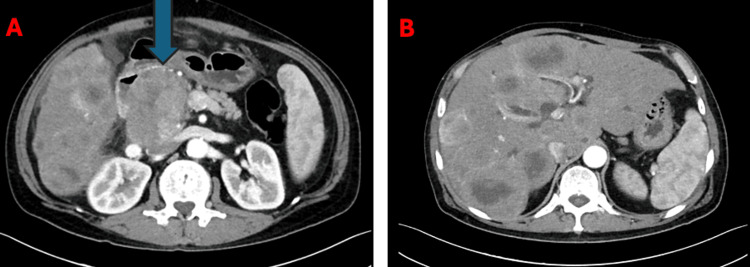
(A) CT of the abdomen showing a large mass in the pancreaticoduodenal groove (blue arrow). (B) CT of the abdomen showing multiple lesions in the liver, suggestive of liver metastases.

A large heterogeneously enhancing mass, measuring 7.8 × 6.3 cm, was identified in the pancreaticoduodenal groove. It caused a significant mass effect on the common bile duct (CBD), resulting in severe upstream biliary dilatation (Figure 1).

Multiple hepatic metastatic lesions of variable sizes were scattered throughout the liver parenchyma. Some lesions exhibited peripheral arterial hyperenhancement, others appeared hypovascular, and a few were hypervascular. The first target lesion was located in the caudate lobe, measuring 3 cm. The second target lesion was identified in segment IV, measuring 6.6 cm. No other distant metastases were observed (Figure 1).

Endoscopic Ultrasound

**Figure 2:**
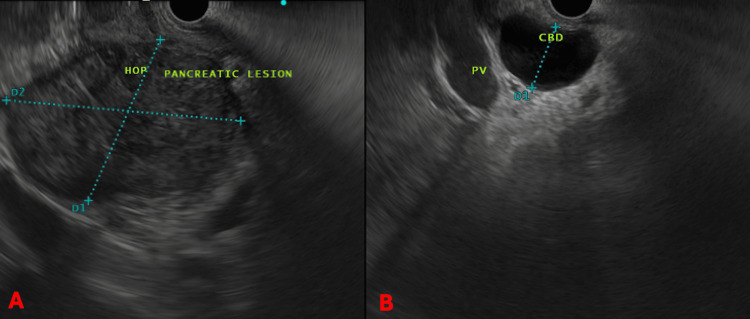
(A) Endoscopic ultrasound (EUS) showing a large mass in the pancreatic head. (B) EUS showing a dilated common bile duct. HOP: head of pancreas; CBD: common bile duct; PV: portal vein.

A round, hypoechoic mass with calcifications and cystic components was visualized in the pancreatic head, measuring 57 × 73 mm at its largest dimension. The mass had well-defined borders and showed signs of invasion into the duodenal bulb. However, an intact interface with adjacent structures suggested no direct invasion (Figure 2).

Fine-needle aspiration (FNA) was performed from the pancreatic head mass (Figure 2).

Endoscopic Retrograde Cholangiopancreatography

**Figure 3:**
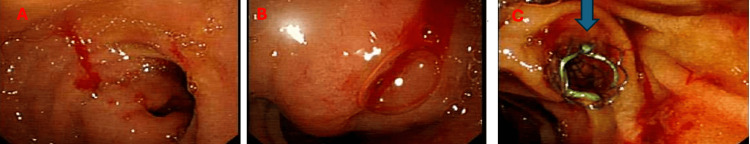
(A, B) Endoscopic retrograde cholangiopancreatography (ERCP) showing moderate biliary stricture with a mass causing obstruction. (C) Stent placement to relieve the obstruction (blue arrow).

Endoscopic retrograde cholangiopancreatography (ERCP) revealed mild dilation of the middle and upper thirds of the main bile duct due to a stricture, with the most significant narrowing measuring 4 mm in width (Figure 3).

**Figure 4:**
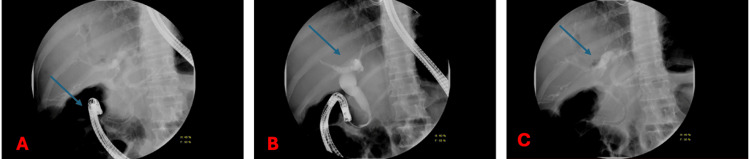
Endoscopic retrograde cholangiopancreatography (ERCP) fluoroscopic images showing successful cannulation (A) and contrast opacification of the biliary tree, with normal biliary anatomy and no evidence of obstruction or filling defects (B, C).

Sludge was present in the bile duct and was cleared using a 12 mm balloon sweep. To relieve the obstruction and improve bile flow, a stent was placed in the biliary tree. The procedure was completed without immediate complications (Figures 3, 4).

Histopathological and immunohistochemical studies (from FNA of the pancreatic head mass)

Microscopy showed malignant spindle cells with nuclear hyperchromasia, marked pleomorphism, frequent mitoses (4/1 HPF) with atypical figures, and areas of necrosis. The tumor cells were positive for CD117 and DOG-1, and negative for CK AE1/AE3, CK8/18, synaptophysin, and chromogranin. These findings are consistent with GIST, favoring a high-risk category.

Management

The patient was started on imatinib 400 mg daily due to the presence of liver metastases and the unresectable nature of the tumor.

Follow-up

**Figure 5:**
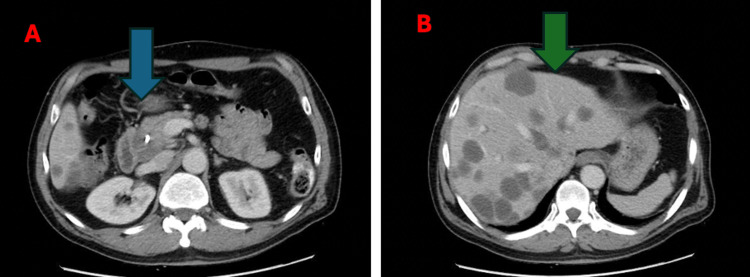
(A) CT of the abdomen shows a significant interval decrease in the size of the previously seen pancreatic mass located in the pancreaticoduodenal groove (blue arrow). (B) CT of the abdomen shows a significant decrease in the size and number of the hepatic metastatic lesions (green arrow).

A CT scan of the abdomen performed at six months showed a significant interval decrease in the size of the previously noted extra-pancreatic mass in the pancreaticoduodenal groove, now measuring approximately 3 × 3 cm, down from the prior measurement of 8 × 7 cm. The mass exhibited reduced enhancement and marked improvement in compression of the CBD and associated upstream biliary dilatation (Figure 5).

There was also a substantial reduction in the size and number of hepatic metastatic lesions. Most lesions showed decreased enhancement, with only a few demonstrating mild peripheral enhancement. The largest metastatic liver lesion, which previously measured approximately 7.5 × 5 cm, had decreased to approximately 3.8 × 3 cm (Figure 5). The patient’s laboratory results returned to normal.

## Discussion

Pancreatic GISTs are exceptionally rare, comprising only 1%-2% of all GISTs, which themselves account for less than 1% of gastrointestinal neoplasms [1,2]. Most GISTs arise in the stomach (60%) and small intestine (30%), with the pancreas being a particularly uncommon extragastrointestinal site [3].

Pancreatic GISTs may present with nonspecific symptoms such as abdominal pain, weight loss, or, less commonly, obstructive jaundice, as seen in this case. Obstructive jaundice occurs when the tumor compresses or invades the biliary tract. Though uncommon, this has been documented in isolated reports [4]. Imaging findings are often nonspecific, and these tumors may be radiologically mistaken for pancreatic adenocarcinoma, neuroendocrine tumors, or solid pseudopapillary neoplasms [8].

Histopathological evaluation is essential for definitive diagnosis. Immunohistochemical staining for CD117 (KIT) and DOG-1 is positive in more than 95% of GISTs [5,9]. Histologic subtypes include spindle cell (most common), epithelioid, and mixed forms. Molecular testing for KIT and PDGFRA mutations further aids in diagnosis, helps guide therapy, and predicts treatment response [5,9].

Surgical resection is the primary curative option for localized disease. Depending on the tumor’s location, pancreatic GISTs may be amenable to organ-preserving approaches [6,7]. In metastatic or unresectable cases, targeted therapy with imatinib mesylate, a tyrosine kinase inhibitor, remains the standard of care. Imatinib has shown high response rates, particularly in tumors with KIT exon 11 mutations, and is also used in neoadjuvant and adjuvant settings to reduce tumor burden or recurrence risk [10,11].

This case highlights the importance of considering pancreatic GISTs in the differential diagnosis of obstructive jaundice and demonstrates the potential of targeted therapy to achieve substantial clinical and radiological improvement, even in advanced disease.

## Conclusions

Pancreatic GISTs, although exceedingly rare, should be included in the differential diagnosis of atypical pancreatic masses, particularly when presenting with obstructive jaundice. Accurate diagnosis relies on targeted imaging, histopathological evaluation, and immunohistochemical confirmation. While surgical resection remains the preferred approach when feasible, the use of imatinib in unresectable, metastatic, or high-risk cases has significantly improved outcomes. This case highlights the importance of early recognition and the role of targeted therapy in achieving meaningful clinical and radiological responses, even in advanced disease stages.
